# Understanding stressors in combination: a continued challenge for human performance

**DOI:** 10.1113/EP092995

**Published:** 2026-03-13

**Authors:** Katrina Hinde, Jennifer Wright, Ella Walker

**Affiliations:** ^1^ Human Performance Team, Defence Science and Technology Laboratory Salisbury UK

**Keywords:** combined stressors, occupational, performance

## Abstract

Personnel within occupational employment (e.g., military/emergency services) are exposed to various stressors concurrently including psychological, cognitive, physical and environmental. Historically, stressors have been considered and studied in isolation which is not representative of reality. Assessing stressors in combination is challenging for researchers owing to the study designs required to explore such interactions. Complex study protocols can lead to logistical challenges and high demands on resources and participants. Very few studies within the literature have been found to explore multiple stressors, although in recent years, this has started to change for the better. Understanding how numerous stressors interact, whether effects on performance are additive, synergistic or antagonistic, is important. Without this, the true impact of stressors will remain unknown, and the health and performance of those within arduous occupational roles may not be optimised. This review aims to (1) explore how different study designs have enabled the exploration of the effects of combined stressors on human performance outcomes in controlled laboratory settings, simulated field studies and field‐based settings, and (2) outline how future research can develop methodologies that study combinations of stressors in occupational roles.

## Abstract (highlights)

- **What is the topic of this review?**
  This review explores how combinations of stressors and their interaction effects have been applied to human performance research.
- **What advances does it highlight?**
  Using evidence from laboratory, simulated‐field and field‐based studies, we provide examples of how studying combined stressors leads to enhanced understanding of human occupational performance. Furthermore, this review outlines how future research can (and should) consider studying combinations of stressors and their interactions, to guide interventions and strategies to mitigate against degradation of cognitive and/or physical performance when stressors are experienced simultaneously.

## 1 INTRODUCTION

Many occupational roles experience a multitude of scenarios that present simultaneous cognitive and physical challenges. Such situations can compromise work capacity and safety unless the stressors are mitigated. For defence and security personnel, the ability to overcome a combination of physically, mentally and environmentally demanding stressors can be essential in determining success. Other occupations that frequently experience combinations of stressors include (but are not limited to) the outdoor and adventure industry, those working in emergency services, the gas and oil industry, and agricultural industry. These occupations expose workers to physically demanding and complex tasks in arduous conditions.

Typically, research in these occupations has investigated stressors and tasks in isolation, and once understood, have presented solutions on how the impact of the stressor can be reduced or optimised for the work conducted. This reductionist approach has rarely progressed further to consider stressors and tasks in combination with others that are presented simultaneously (for example, the combined impact of cognitive load and thermal strain). Although the reductionist approach has allowed a baseline level of understanding of the stressors, the ecological validity of this research when applied to occupational contexts is likely reduced, due to the interplay of additional stressors. As such, the *true* impact of stressors on occupational human performance remains equivocal.

**FIGURE 1:**
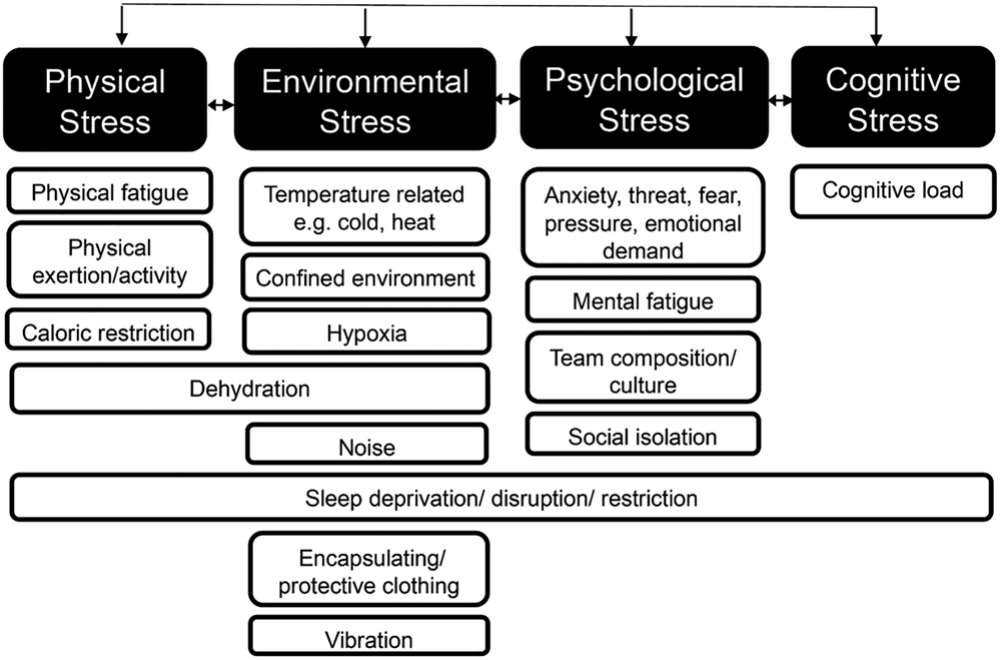
Examples of physical, psychological, environmental and cognitive stressors.

The term ‘stressor’ can be defined in many ways and as a result can lead to different outcomes and assessment methodologies. To avoid confusion, the term stressor in this review will be defined as physical, psychological, cognitive and environmental circumstances that disrupt homeostasis (Chrousos, 2009). Examples of stressors experienced in occupational roles are presented in Figure 1.

The need for research to progress beyond considering stressors singularly has been acknowledged in literature and reflects a systems approach (i.e., exploring interactions between components). Tipton (2012) highlighted that particularly in environmental physiology, stressors are often encountered in combination (noting that cold and hypoxia usually co‐exist, as do cold and hyperbaria [increased barometric pressure, which usually involves a high delivery of oxygen]) but they are rarely studied together. Furthermore, the response to some stressors only occurs (or are more likely to occur) when experienced alongside other stressors (Tipton, 2012). For example, Golja et al. (2004) observed that when cold stimuli were applied via a thermosensitivity testing device, hypoxia increased the cutaneous threshold for the sensation of cold when compared to normoxia. However, hypoxia had no effect on the sensation of warmth when a warming stimulus was presented. The practical application of this research was that behavioural thermoregulatory actions could be compromised in hypoxic–cold conditions, and thus individuals should take care to ensure the temperature of the extremities does not fall below the range at which cold injury can occur. These results demonstrate how understanding stressors in combination can improve our mechanistic understanding of the human body, and how changes to working practices could improve both health and performance.

The challenge of conducting combined stressor research is compounded by how laboratory‐based research is not always translational to ‘real world’ situations. Although laboratory research can set the foundations for conducting field research and vice versa, often retrospectively fitting the results of laboratory research to occupational roles may not always be appropriate. This is because many occupational roles and tasks involve volatile and constantly evolving situations, which cannot be replicated in laboratory research. Nevertheless, research designs that incorporate more realistic operating environments and have methods for separating and/or controlling for multiple effects is essential to elucidate the possible interaction effects of multiple stressors. In order to isolate the individual effects of a combined stressor environment, one would ideally use a crossover design (i.e., ensuring the same participants are used for all conditions) with appropriate washout periods, and consider the need for counterbalancing and randomised exposure to stressors. In addition, studies like these would likely need to incorporate familiarisation (to control for, or identify, any learning or training effects), and put consideration toward any other covariates that need accounting for. If such design elements are not possible, then advanced statistical models may be used to explore variance in different ways.

The lack of combined stressor research has been postulated by Tipton (2012) to be driven by numerous factors including funding, individual interest, facility availability and pigeonholing of expertise into singular areas. In more recent years, however, the importance of multi‐ and inter‐disciplinary approaches to research has led to an increased number (albeit still low) of scientific publications in this area. It is without argument that conducting combined stressor research is complex and costly, partially explaining why the number of combined stressor studies remains low.

### 1.1 Interaction of stressors concept

To aid the methodological challenges of researching stressors in combination, Lloyd and Havenith (2016) proposed that the interaction between stressors can be generally categorised based on the effect they have on each other. These interactions were termed additive, synergistic (hyper‐additive) or antagonistic (subtractive or hypo‐additive). An overview of these terms is presented as follows:

1. Additive stressors indicate one variable has no differential effect in the presence of the other variable, that is, the effect of the combination of stressors A + B is equal to the effect of stressor A + B separately.
2. A synergistic interaction indicates that the overall effect is significantly greater than the additive effect.
3. An antagonistic, or hypo‐additive, interaction indicates where the net effect is lower than the additive effect.

The categories can be further subdivided to include: (1) ‘the most severe component’, whereby the severity of one stressor cancels out the impact of another stressor; (2) relative addition, where additive and antagonistic factors interact (in this, the percentage impact remains the same in the presence of another stressor); and (3) ‘impact nullification’, where the combined impact of stressors is lower than the individual impact.

Lloyd and Havenith (2016) stated that the mechanisms by which a stressor impacts the human body dictates the category in which the stressor interaction occurs. Broadbent (1963) and Lloyd et al. (2015) noted that stressors with independent mechanisms often have additive effects, whereas stressors that are mechanistically similar are likely to have synergistic or antagonistic effects. It has also been suggested that the interaction categories between stressors can be based on how much of an impact a stressor has on an individual (i.e., the magnitude), and the resulting influence on performance (Lloyd & Havenith, 2016). Within this framework, mild stressors produce additive effects; however, as the impact of a stressor increases, the interaction type may shift to antagonistic, and can lead to the most severe component being the most influential on performance. From understanding how stressors interact, we can improve how we mitigate their impact on health, task performance and safety.

### 1.2 Aim

This review aims to explore how different study designs have enabled the exploration of the effects of combined stressors on human performance outcomes in controlled laboratory settings, simulated field studies and field‐based settings. This will enable us to outline how future research can consider studying combinations of stressors within occupational roles. This review will not seek to summarise the literature related to the effect of combined stressors on human performance (e.g., physical, cognitive, occupational) due to the vast number of combinations relevant to occupational roles.

## 2 FIELD‐BASED STUDIES

True field studies are complicated and complex to conduct as they occur in real‐world environments, but consequently, they reflect real‐world context, behaviours and responses. In occupational settings, the challenge is primarily due to the environmental and organisational context in which individuals work. For example, some environments firefighters work in are hazardous to researchers untrained for that scenario. Additionally, the scientific equipment selected to monitor or assess the personnel may fail or introduce risk. Military training exercises or operations have specific aims and objectives which research should not detract from. This limits access to participants, causing variation in testing/sampling times, or restricting the amount of scientific experimentation that can occur. As such, most field studies often become purely observational as researchers are unable to manipulate any stressors or conditions.

In a classic field study, Opstad et al. (1978) built cognitive assessments into a training course at the Royal Norwegian Military Academy (i.e., they were tested each morning). This enabled the authors to track when the effect of the multi‐stressor environment started to take effect. Understanding this temporal dimension is assistive to military practitioners, for example, allowing them to understand when interventions could best be deployed. Field studies often have limited time for data capture, irrespective of design. Following military operations, training and exercises, studies have shown reductions in body mass, physical performance, hormonal disturbances (Hamarsland et al., 2018; Lieberman et al., 2016; Marrao et al., 2005) and cognitive performance (Erez et al., 2024; Lieberman et al., 2016; Marrao et al., 2005). In the study by Hamarsland et al. (2018), access to participants was not possible during the extremely demanding training week, nicknamed ‘Hell Week’ in the Norwegian Special Forces selection course. As such, measurements were only able to be performed pre, immediately post and at various time points during a 2‐week recovery period. Consequently, analysis was unable to determine when impairments became significant. As stated earlier, identifying the time point at which performance decrements start to occur could inform preparation or mitigation strategies.

A second classic field study by Rognum et al. (1986) showed that sleep deprivation was a major factor influencing physical performance and energy deficit/sustained physical activity had minimal contribution. The study design enabled the observation of 24 male soldiers during a field exercise combining sleep deprivation (<2 h over 107 h), caloric deficit and sustained physical activity. To investigate whether energy deficit significantly impacted performance, the investigators were able to manipulate diet; some soldiers received a high energy intake (8000 kcal per day, *n* = 9), while the rest were in energy deficit (1500 kcal per day). Taken together this study showed that the soldiers’ physical performance was impaired over time in both groups, and interestingly a high energy intake did not lessen the level of impairment shown. The study design by Rognum et al. (1986) went some way towards understanding combined stressors, as it showed that in this instance, caloric deficit had minimal effect on overall outcome, highlighting to the military practitioners that a multifactorial intervention would be required to improve performance, that is, supplying more calories alone would not be sufficient.

A novel field study conducted during winter military training in Norway characterised the effect of a multi‐stressor environment (extreme cold, sustained physical exercise involving heavy load carriage, energy deficit) on physical performance, energy balance and whole‐body protein turnover (Margolis et al., 2014). Measures of energy expenditure and intake were taken in the field throughout the cold weather training using doubly labelled water and analysis of discards, respectively. Whole‐body protein turnover was measured via ingestion of ^15^N‐glycine and urine was collected over a 10 h period. This methodology has been highlighted as being a practical method effective for measuring protein turnover in military field research (Hinde et al., 2021). This study enabled the overall effect of the stressors on physiological and performance measures. Margolis et al. (2014) reported that although protein intake was within recommendations, a negative whole‐body protein balance was evident, suggesting that protein intake during winter military training may need to be higher. As a result, Margolis et al. (2016) investigated the effect of providing supplemental rations with protein or carbohydrate in attenuating negative balances (both energy and protein) during Arctic military training. Although it was reported that soldiers with the highest energy intake achieved net protein balance, physical performance was not measured and thus the impact of the intervention was not fully evaluated. These studies demonstrate that characterising the effect of stressors initially in the field enabled a targeted countermeasure to be employed in a follow‐on study.

The nature of the training/exercises/operations mentioned above dictate the design of the study as it is important for occupational research to have minimal impact on courses that are ultimately career defining. There must be consideration for data quality and quantity versus participant burden and invasiveness of the measures. Due to these considerations the one limitation all these studies have in common is the inability to distinguish the individual effects each stressor was having on performance. These studies can quantify the combined effect of all stressors involved on overall performance (cognitive, physical or military), but identifying whether stressors are additive, or whether a particular stressor dominates the response is key to effectively and efficiently identifying mitigation strategies.

### 2.1 Simulated‐field studies

Akin to field‐based studies, some simulated sustained military operation studies (Conkright et al., 2021; Keramidas et al., 2018; Lieberman et al., 2006, 2016; Nindl et al., 2002, 2006), whilst high in ecological validity, were still unable to separate stressors. In the cited studies, participants were exposed to simulated operations or training exercises, which involved up to four combined stressors. Comparisons were made to either a control trial, or a baseline period during which physical and psychological demand was low, and individuals experienced habitual sleep and feeding habits. As such, the authors could quantify the overall impact on military, physical and/or cognitive performance, but were unable to identify whether it was a combination of stressors in equal measures impacting performance or whether any of the stressors were more influential than others. In most instances, to tease out which stressors were most dominant was not the main purpose of the research, and the lack of inclusion of the effects of isolated stressors, as well as together, may be attributable to the fact that the study design would be experimentally and practically challenging. Studies using simulated conditions (Conkright et al., 2021; Keramidas et al., 2018; Lieberman et al., 2006, 2016; Nindl et al., 2002, 2006) do, however, show that the impact of a military operation can be successfully replicated/simulated using a mixture of laboratory and field‐based settings, and thus, greater experimental control can be employed. Demonstrating the benefit of incorporating measurements during combined stressor events, Lieberman et al. (2016) were able to observe changes in stress hormones and heart rate associated with simulated captivity and interrogation, despite low physical activity. Performing measurements at multiple time points – baseline (the week prior to the training exercise), immediately prior, during and immediately following the captivity phase of training – allowed researchers to track response recovery from peak values. Lieberman et al. (2006) concluded that simulation studies could enable quantitative and interactive effects of various stressors to be investigated.

To address some of the challenges mentioned above associated with field studies, simulated field studies trade some of the ecological validity for control and bridge the gap between laboratory studies and real‐world complexity. Several simulated field studies have successfully explored changes in performance in multi‐stressor environments by separating and combining stressors. To illustrate this, Smith et al. (2016) investigated the ability of firefighters to self‐monitor their performance during a simulated fire‐ground tour (involving multiple circuits of physical work, non‐work, cognitive testing and rest) during sleep restriction, heat exposure and a combination of both. The authors, through the study design, were able to explore the isolated effects of heat and sleep restriction and their interactive effects. Participants were split into four groups: (1) control (8 h sleep opportunity and ambient temperatures 18–20°C); (2) sleep restriction (4 h sleep opportunity and ambient temperatures 18–20°C); (3) hot (8 h sleep opportunity and ambient temperatures 33–35°C [day], 23–25°C [night]; and (4) sleep restriction and hot (4 h sleep opportunity and ambient temperatures 33–35°C [day], 23–25°C [night]). The study did not utilise a repeated measures design (i.e., different participants experienced different experimental conditions), and therefore it was acknowledged that participants may vary according to cognitive performance and that the incremental impact of fatigue could vary between individuals. As such, reciprocal reaction times on the Psychomotor Vigilance Task were standardised to individual performance at baseline, and random slopes within participants included in subsequent linear mixed effects models. The authors concluded that the combination of heat exposure and sleep restriction led to greater reductions in cognitive performance and lower self‐assessments of performance compared to the effect of individual stressors or control conditions (Smith et al., 2016). Due to the way in which the data were presented, it is difficult to determine the interaction effect in the way Lloyd and Havenith (2016) describe. Nevertheless, this study successfully simulated a 3‐day firefighter deployment using an ecologically valid battery of physical tasks.

Another example of a study (1) using a simulated deployment, (2) taking measurements throughout and (3) aiming to explore the interactive effects of multiple stressors is the study by Beckner et al. (2023). This focused on the effect of energy balance on cognitive performance during a 72‐h simulated sustained military operation. Whilst both sleep restriction and energy deficits are commonly experienced stressors during military operations, total sleep deprivation has been shown to have a greater effect on cognitive performance than total energy deficit during survival training (Ståhle et al., 2011). However, energy deficit has been shown to impair high level cognitive processes such as task‐switching and response inhibition (Giles et al., 2019). Reducing energy deficit was identified as a long‐term goal of military nutrition research in the US and the impact of an energy deficit on cognitive performance during military activity was unclear (Beckner et al., 2023). The authors concentrated on stressors that were more easily manipulated (i.e., the physical and psychological demand are to some degree uncontrollable). As such, energy deficit was the main focus of this study as it was believed that strategies to reduce energy deficits may be more feasible for military populations during sustained operations compared to strategies aimed at reducing physical activity or sleep loss (Beckner et al., 2023). The decision to focus on modifiable stressors is commended and whilst acknowledging the challenges associated with increasing the amount of sleep in active‐duty military personnel, the effectiveness and feasibility of strategies have been explored (Keramidas et al., 2018a; Mesas et al., 2023; Ritland et al., 2019). Beckner et al. (2023) had two experimental conditions: (1) energy balance (provided with food to meet 100% of predicted total daily energy expenditure and restricted sleep of 4 h a night) and (2) energy deficit (provided with food to meet 45% of predicted total daily energy expenditure, and restricted sleep of 4 h a night). An experimental condition that had a greater sleep opportunity would have enabled the independent effect of energy deficit (without the influence of sleep restriction) to have been quantified. This would provide insight into whether improving sleep provision, or reducing the effects of sleep restriction, could influence whether energy deficit, alongside other stressors (e.g., strenuous physical activity) impacts performance. Furthermore, as acknowledged by the authors, the study was significantly limited by sample size (*n* = 7 for cognitive performance and mood variables) and only included male participants. As such, caution should be applied to the conclusions. A larger research study incorporating the effects on physical, cognitive and task performance would contribute greatly to the evidence base.

## 3 LABORATORY‐BASED COMBINED STRESSOR RESEARCH

The ability to manipulate environmental conditions via an environmental chamber, or to regulate cognitive stimuli (e.g., to create differing levels of cognitive load or fatigue), enables multiple stressors to be explored. As such, there are a greater number of (but still relatively few) laboratory‐based studies exploring combined stressors and their interactions. What these studies lack in ecological validity, they make up for in experimental control and are logistically simpler by design. Laboratory‐based research has been able to take multi‐stressor environments and conduct repeated measures study designs examining the effect of the stressors when isolated and combined.

**FIGURE 2:**
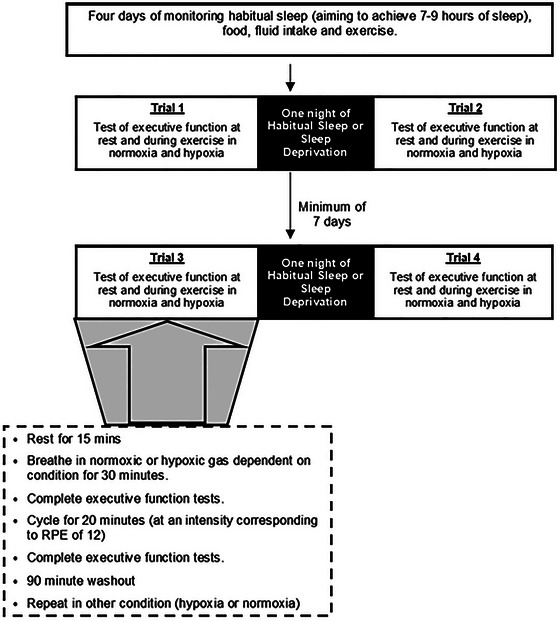
Study design employed by Williams et al. (2024). An example of a full trial protocol is shown within the dashed box. Trials 1 and 3 were conducted to ensure there were no differences in executive function task performance at rest between the three trials without sleep deprivation. Data from trials 2 and 4 were used for analysis. RPE, rating of perceived exertion.

A good case study is that by Williams et al. (2024) who explored the effect of combined sleep deprivation, hypoxic exposure and acute exercise on executive function. Previous work has shown that individuals who frequent high altitude environments have disturbed sleep (de Aquino Lemos et al., 2012; Jafarian et al., 2008), whilst exposure to high altitude and sleep deprivation has been shown to reduce cognitive performance independently (Killgore, 2010; Lim & Dinges, 2010; McMorris et al., 2017). In contrast, acute bouts of exercise have been shown to improve cognitive performance in normoxia (Chang et al., 2012) and hypoxia (Jung et al., 2020). Williams et al. (2024) stated that suboptimal functioning of the prefrontal cortex could contribute to reduced cognitive performance in situations of both hypoxia and sleep deprivation, and there may be a protective role of physical exercise for tasks that require the prefrontal cortex. The methodology employed by Williams et al. (2024) involved a within‐participant, repeated measures controlled crossover study (Figure 2) where the order of hypoxia/normoxia and total sleep deprivation (TSD) or habitual sleep was counter‐balanced. This design enabled the isolated and combined effects of TSD, acute hypoxia and physical activity to be explored. To do so, a series of three two‐way repeated measures ANOVAs were conducted. The authors could have employed a three‐way (environment [hypoxia, normoxia] × sleep status [habitual, TSD] × exercise [rest, exercise]) repeated measures ANOVA to identify whether the third factor plays a role in the interaction between the first two, or a random slopes analysis like Smith et al. (2016), to incorporate variance for individual participants. However, the analysis conducted still provides information on different combinations of stressors on executive function performance.

**FIGURE 3:**
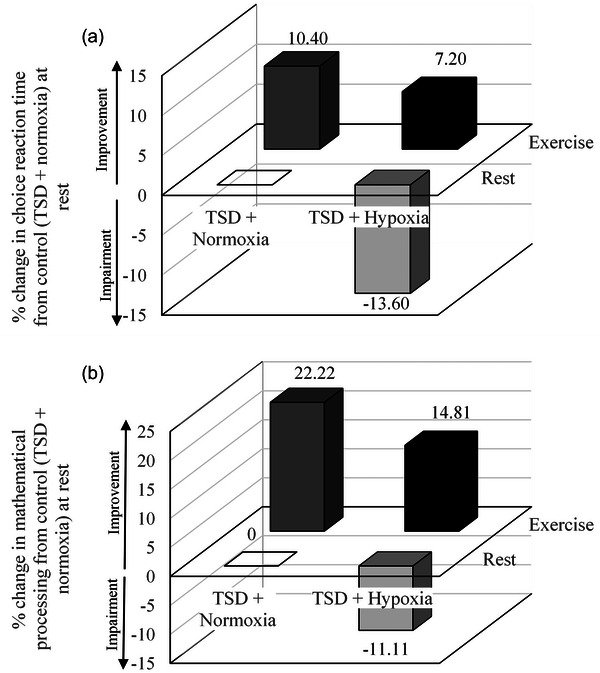
Interactions observed by the authors using supplementary data (Williams et al., 2024). (a) Percentage change in choice reaction time performance compared to ‘control’ conditions (TSD + normoxia at rest). (b) Percentage change in mathematical processing compared to ‘control’ conditions (TSD + normoxia at rest).

Using supplementary data provided by Williams et al. (2024), we were able to compute the interaction effects of sleep status and environment during both rest and exercise and these are shown in Figure 3. For choice reaction time, a synergistic (hyper‐additive) interaction effect is reported (*P* = 0.039), whereby exercise provides a positive effect on cognitive performance when sleep deprivation is experienced in normoxia. Individuals experiencing sleep deprivation in hypoxia display significant reductions in choice reaction time when resting; however, the addition of acute physical activity improved cognitive performance to levels just below that at sea level (Figure 3a). Similarly, for mathematical processing, the interaction was also synergistic (*P* = 0.042) (Figure 3b).

Limitations of the study by Williams et al. (2024) include the potential for lack of control during sleep deprivation trials. Most of the study took place during the outbreak of COVID‐19 and as a result, participants undertaking the sleep deprivation were unable to undertake the sleep deprivation nights at the university laboratory, and it was consequently conducted unsupervised at the participants’ home. The researchers requested the participants to send a text message every hour to record their being awake. However, sleep could have taken place in between these hourly messages, although accelerometery data did not indicate that this was the case. Nevertheless, the researchers were working in a new, unprecedented situation and should be commended for being able to maintain and continue their research. Furthermore, this also shows the feasibility of conducting sleep debt protocols in the participant's home, reducing costs and demand on research staff. In addition, the hypoxic exposure was very acute (<60 min), and thus the effect of a chronic exposure, which may be more ecologically valid, was not investigated. And finally, due to different reasons, only 9 out of the 12 participants were able to complete all conditions, and thus sample size is small throughout.

**FIGURE 4:**
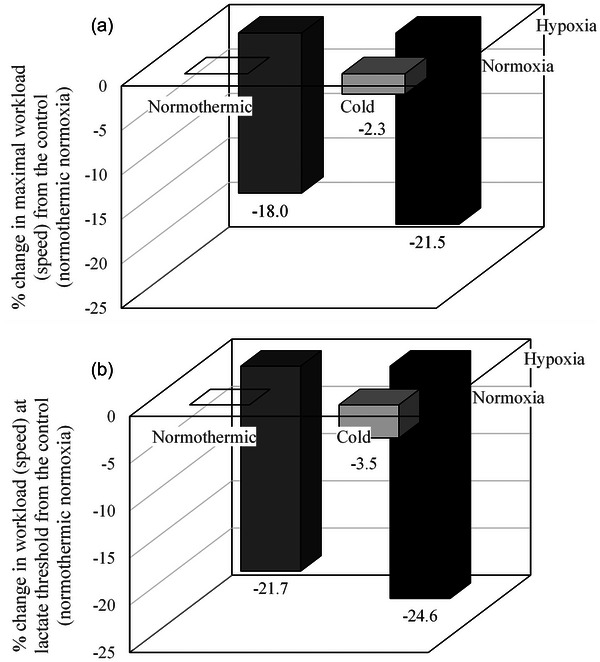
Additive effects observed by the authors using data from Callovini et al. (2024). Percentage change in maximal workload (speed) (a) and lactate‐threshold (b) workload (speed) compared to ‘control’ conditions (normothermic normoxia).

Tipton (2012) noted that high altitude commonly exists in combination with low ambient temperatures, but few laboratory studies explore both together. Another recent example of a laboratory‐based study investigating combinations of stressors comes from Callovini et al. (2024, 2025) who enabled further insight into dynamic interaction characteristics by conducting a repeated measures, crossover laboratory study which involved participants performing a submaximal treadmill test to exhaustion in different environments. In both studies, conditions were (1) normothermic normoxia (18°C, $F_{iO_{2}}$ = 0.21) (N); (2) normothermic hypoxia (18°C, $F_{iO_{2}}$ = 0.13, representing ∼3500 m) (H); (3) cold normoxic (−20°C) (C); and (4) cold hypoxia (CH). A series of two‐way (temperature × $F_{iO_{2}}$) and three‐way repeated measures ANOVAs (temperature × $F_{iO_{2}}$ × intensity) were performed to determine changes in performance, physiological and perceptual responses (Callovini et al., 2024). Whilst measures of lung function and respiratory muscle strength were measured in Callovini et al. (2025) and generalised estimating equation analysis was used due to missing data, Callovini et al. (2024) found significant reductions in workload (speed) due to changes in $F_{iO_{2}}$ (*P* < 0.001) and reduced ambient temperatures (*P* = 0.001) but no significant interaction effect (*P* = 0.25). The study abstract noted an additive effect for changes in relative workload (speed) at maximum and sub‐maximum exercise intensities, yet this additive effect was not elaborated on within the results section of the study. Using values provided by Callovini et al. (2024), Figure 4 was created for interactive effects. The combination of cold and hypoxia led to a reduction in workload (speed) that was similar to the sum of reductions seen in hypoxia and cold independently (i.e., additive). Figure 4 also shows that the majority of the reduction in workload during HC is attributable to changes in $F_{iO_{2}}$. The study design showed that cold exposure in this instance, has minimal effect.

This study was limited by using only male participants, and thus further investigation is required to establish if sex differences in responses exist. Furthermore, continuous breath‐by‐breath gas analysis was not conducted in the study by Callovini et al. (2024) due to challenges presented by the cold ambient temperatures and the operating temperatures of the online gas analysis system. Consideration to other strategies could be given, whereby the online gas analysis system could be housed outside of the environmental chamber and insulated sample lines fed in through openings in the chamber walls (Hinde et al., 2018), or Douglas bags used. Furthermore, temperature measurements (e.g., skin and core body temperature) were not conducted, making it unclear to what extent participants experienced cold strain or how it influenced outcomes. Participants wore extreme weather clothing and were allowed to adjust their clothing as they exercised, and so the authors hypothesised that any impact of the cold on physiological parameters or exercise performance may primarily be attributable to airflow limitation rather than changes in core body temperature (Callovini et al., 2024).

**FIGURE 5:**
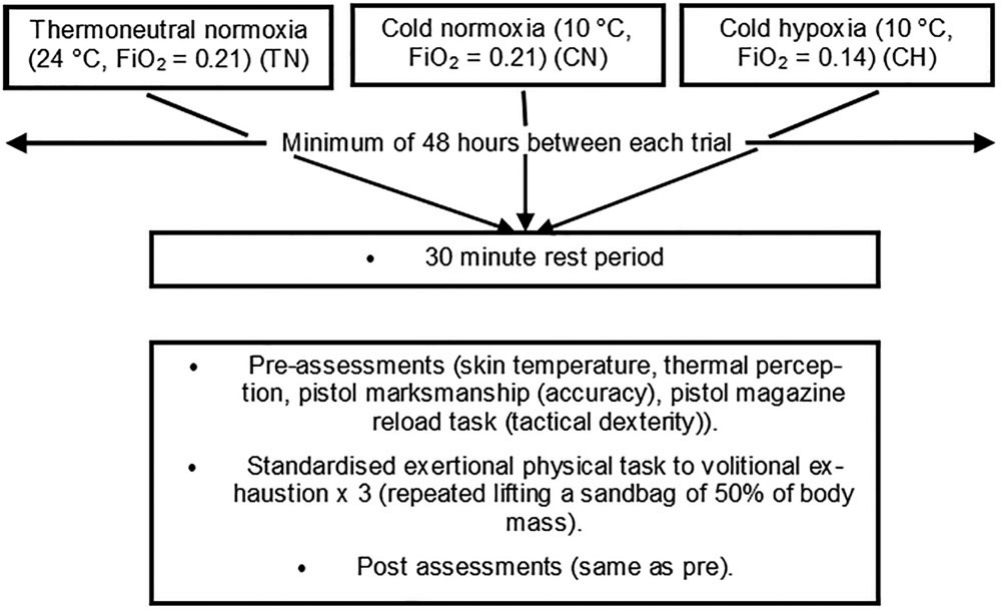
Study design employed by Salmon et al. (2023).

In a final case study of laboratory‐based combined stressor research more relevant to some occupational roles, Salmon et al. (2023) noted that, to their knowledge, no previous research, had examined the combined effects of cold and hypoxia on tactical performance. As a result, they designed a study to investigate the individual and combined effects of cold exposure, hypoxia and physical exercise on shooting performance and subjective thermal perception. The study took place in a custom‐built environmental room and participants took part in a randomised crossover study design with three environmental conditions (Figure 5).

Data were analysed using a mixed effects model (fixed effects; conditions [3] and time [2: pre, post]). By separating out the stressors and adding in a combined condition, the authors were able to show that prior to any physical activity, pistol accuracy was ∼14% lower in CN than TN, but post‐physical activity, accuracy was not significantly affected by the cold. Interestingly, there were no significant differences in pistol accuracy between CH and TN prior to physical activity, but post activity, pistol accuracy was reduced by 17% in CH when compared to TN and 10% when compared to CN. Reload time was significantly slower in both cold conditions. The study concluded that resting/minimal activity combined with cold exposure reduced shooting accuracy and tactical dexterity, but these improved immediately following physical activity, which was found to increase thermal perception also. Hypoxia, however, when experienced alongside the cold and with the addition of physical exertion led to decrements in marksmanship (Salmon et al., 2023). The independent effect of hypoxia on marksmanship was not explored in this study (i.e., a thermoneutral hypoxia condition), and although there are few places that one can experience high altitude without concomitant low ambient temperatures, such an experimental condition could provide additional information on hypoxia‐specific changes that could influence performance, perception and thermoregulation.

## 4 Design and analysis considerations for future research

### 4.1 Considerations for both field and laboratory studies

It has been noted that designing and conducting repeated measures design protocols to tease out interaction effects is demanding (for both participant and researchers), costly and can be logistically challenging. To address this and to minimise the burden on both researcher and participant, consideration and prioritisation could be given to stressors that can be more easily manipulated, that is, that we have control over, and therefore can be mitigated against. For example, the combination of physical stress, psychological/mental stress, energy deficit and sleep disruption is the most common combination of stressors experienced in occupational roles, especially military field exercises, training and operations. The demanding physical and psychological aspects of a military exercise or operation are unlikely to be adjusted, owing to the overarching objective of the training or deployment. The severity of physical and psychological stressors may be determined largely by the presence of other stressors, such as sleep disruption or energy deficit, making them both independent and dependent factors. As such, a study design focusing on manipulating the sleep disruption and/or energy deficit stressors could reduce the number of repeated trials required.

Availability of an adequate number of volunteers to sufficiently power human performance research studies can be challenging. As a result, the number of variables that are measured is often restricted. This is especially true for field‐based and simulated‐field research, as it is paramount that the research does not interfere with the training/exercise/deployment. Researchers working with occupational groups (e.g., firefighters, military) should work with the relevant organisations to facilitate access to a pool of volunteer participants and highlight the benefit of scheduling more time with them in their work schedule. Collecting robust data from a greater range of variables could provide additional and more meaningful insight (e.g., superior biomarkers identified, refinement of an intervention) and enable solutions to problems, or improved working practices to be found.

Despite the need for ecologically valid studies, there is a large requirement for laboratory studies in combined stressor research due to the ability to control and standardise stressors. This level of control enables researchers to assess if changes in performance are due to the contributions of individual stressors, the mechanisms behind these stressors, and the potential influence of individual day‐to‐day variability of the person, environment and tasks conducted. This was highlighted in a recent systematic review by Moore et al. (2025) which explored the cumulative effect of consecutive days of prolonged physical activity or work on physical performance and heat strain. A significant proportion (48%) of the down‐selected studies (*n* = 16) were excluded from the synthesis, despite having high ecological validity. They were excluded on the basis that it was not possible to determine whether changes in performance or heat strain were due to a cumulative effect of exposure to the heat, or between‐day differences in environmental conditions/exercise performed, as conditions were not/could not be standardised across the days. Furthermore, standardised laboratory studies enable other researchers to compare results.

Finally, when considering the human reaction to physical, psychological and environmental stress, it is likely that where research does not consider the individual response to stressors, the effect on individual cognitive, physical or task performance could be masked within the mean values. Future research should therefore seek to report both mean and individual effects of the stressors assessed.

### 4.2 Sex differences

Whilst reviewing the literature, it became increasingly apparent that there is a lack of sex diversity within combined stressor research, which largely has sports science (Costello et al., 2014; Cowley et al., 2021) and military origins (O'Leary et al., 2023). Whilst it is difficult to explain a specific reasoning for the sex bias in research studies, it is likely a combination of circumstances including lower proportions of women working in some physically demanding occupations such as the Armed Forces. It is perceived that recruiting enough women into research to produce sufficiently statistically powered studies is difficult (Kolstoe et al., 2023). In addition, methodological considerations such as factoring in the menstrual cycle, method of contraception or the hormonal status of women was previously deemed too difficult, expensive or beyond the scope of the work (Kolstoe et al., 2023). Reasons for exclusion of participants relating to practicality and resource are no longer acceptable (Kolstoe et al., 2023). Moreover, practical guidance is now available, which highlights the considerations needed to be taken when employing women as participants in research, in an attempt to promote the inclusion of women and generate female‐specific data (Elliott‐Sale et al., 2021). In addition, in areas where new research has been conducted (e.g., muscle protein anabolism), it has been suggested that pre‐menopausal female participants should not be excluded from scientific research on the basis that hormonal status or use of an oral contraceptive pill could be a confounding factor (Colenso‐Semple et al., 2025; Colenso‐Semple et al., 2024). Future research should include males and females and where no previous research has been conducted using females, research should establish whether there are differences between sexes and how this may influence stressors when encountered in combination.

### 4.3 Climate change

It is accepted by the scientific community that climate change has and will continue to result in an increased number of extreme weather events, and that these events will become more frequent in areas that already experience them, but also in new areas (Stott, 2016). Response activities involving occupational employees (such as providing emergency relief to areas impacted by climate events) are likely to become more prevalent as the impact of climate change increases (Applebaum et al., 2016). Secondly, the increasing number of individuals (both military and non‐military) working in areas of cold climates or climate uncertainty means human performance in these regions may become increasingly important, for example, as the melting of sea ice in polar regions opens up potential transport links (Lynch et al., 2022). As such, it is recommended that future research focuses more on extreme climates (such as heat) and combinations of other stressors (e.g., psychological/mental stress, cognitive load, physical stressors, sleep disturbance).

Finally, there is a requirement for research to consider the impact of other stressors on the effectiveness of an intervention. Typically, participants begin well‐rested in interventional research, having had no caffeine, in a state of energy balance and having avoided strenuous exercise. Whilst this is required to minimise confounding factors, it generally does not realistically reflect the end user population. Whether environmental conditions, chronic energy deficit, sleep restriction/deprivation or cumulative effects of physical stress alter the level of effectiveness of an intervention is relatively unknown. If an intervention is shown to be beneficial for performance, additional research could consider whether confounding factors influence its effectiveness.

## 5 CONCLUSION

In summary, relatively few studies (laboratory, simulated‐field and field‐based) have explored stressors in combination, although more recently there has been an increase in laboratory studies. Determining whether interaction effects exist and what category they classify as (e.g., additive, synergistic or antagonistic) is key to understanding which mitigation strategies will be most effective when encountering a particular combination of stressors. Future research should: (1) seek to ensure males and females are considered; (2) establish first whether interaction effects can be obtained in a laboratory environment (preferably utilising a simulated event; once established, translating findings to the field is vital); (3) focus on determining the effects of modifiable stressors on performance; and (4) focus on combinations of stressors involving hot and cold ambient temperatures for reasons associated with climate change.

## AUTHOR CONTRIBUTIONS

Katrina Hinde: Conceptualisation, writing, edits, and figures. Jennifer Wright: Conceptualisation, writing and editing. Ella Walker: Conceptualisation, writing and editing. All authors have read and approved the final version of this manuscript and agree to be accountable for all aspects of the work in ensuring that questions related to the accuracy or integrity of any part of the work are appropriately investigated and resolved. All persons designated as authors qualify for authorship, and all those who qualify for authorship are listed.

## CONFLICT OF INTEREST

None declared.

## References

[eph70243-bib-0001] Applebaum, K. M. , Graham, J. , Gray, G. M. , LaPuma, P. , McCormick, S. A. , Northcross, A. , & Perry, M. J. (2016). An overview of occupational risks from climate change. Current Environmental Health Reports, 3(1), 13–22. 26842343 10.1007/s40572-016-0081-4

[eph70243-bib-0002] Beckner, M. E. , Lieberman, H. R. , Hatch‐McChesney, A. , Allen, J. T. , Niro, P. J. , Thompson, L. A. , Karl, J. P. , Gwin, J. A. , Margolis, L. M. , & Hennigar, S. R. (2023). Effects of energy balance on cognitive performance, risk‐taking, ambulatory vigilance and mood during simulated military sustained operations (SUSOPS). Physiology & Behavior, 258, 114010. 36349660 10.1016/j.physbeh.2022.114010

[eph70243-bib-0003] Broadbent, D. (1963). Differences and interactions between stresses. Quarterly Journal of Experimental Psychology, 15(3), 205–211.

[eph70243-bib-0004] Callovini, A. , Fornasiero, A. , Savoldelli, A. , Decet, M. , Skafidas, S. , Pellegrini, B. , Bortolan, L. , & Schena, F. (2024). Independent, additive and interactive effects of acute normobaric hypoxia and cold on submaximal and maximal endurance exercise. European Journal of Applied Physiology, 124(4), 1185–1200. 37962573 10.1007/s00421-023-05343-9 PMC10955012

[eph70243-bib-0005] Callovini, A. , Fornasiero, A. , Savoldelli, A. , Dorelli, G. , Decet, M. , Bortolan, L. , Pellegrini, B. , & Schena, F. (2025). Combined effects of normobaric hypoxia and cold on respiratory system responses to high‐intensity exercise. Experimental Physiology, 110(12), 1892–1903. 40349318 10.1113/EP092468 PMC12665948

[eph70243-bib-0006] Chang, Y.‐K. , Labban, J. D. , Gapin, J. I. , & Etnier, J. L. (2012). The effects of acute exercise on cognitive performance: A meta‐analysis. Brain Research, 1453, 87–101. 22480735 10.1016/j.brainres.2012.02.068

[eph70243-bib-0007] Chrousos, G. P. (2009). Stress and disorders of the stress system. Nature Reviews Endocrinology, 5(7), 374–381. 10.1038/nrendo.2009.106 19488073

[eph70243-bib-0008] Colenso‐Semple, L. M. , McKendry, J. , Lim, C. , Atherton, P. J. , Wilkinson, D. J. , Smith, K. , & Phillips, S. M. (2024). Menstrual cycle phase does not influence muscle protein synthesis or whole‐body myofibrillar proteolysis in response to resistance exercise. The Journal of Physiology, 603(5), 1109–1121. 39630025 10.1113/JP287342 PMC11870050

[eph70243-bib-0009] Colenso‐Semple, L. M. , McKendry, J. , Lim, C. , Atherton, P. J. , Wilkinson, D. J. , Smith, K. , & Phillips, S. M. (2025). Oral contraceptive pill phase does not influence muscle protein synthesis or myofibrillar proteolysis at rest or in response to resistance exercise. Journal of Applied Physiology, 138(3), 810–815. 40013418 10.1152/japplphysiol.00035.2025

[eph70243-bib-0010] Conkright, W. R. , Beckner, M. E. , Sinnott, A. M. , Eagle, S. R. , Martin, B. J. , Lagoy, A. D. , Proessl, F. , Lovalekar, M. , Doyle, T. L. , & Agostinelli, P. (2021). Neuromuscular performance and hormonal responses to military operational stress in men and women. The Journal of Strength & Conditioning Research, 35(5), 1296–1305. 33780395 10.1519/JSC.0000000000004013

[eph70243-bib-0011] Costello, J. T. , Bieuzen, F. , & Bleakley, C. M. (2014). Where are all the female participants in Sports and Exercise Medicine research? European Journal of Sport Science, 14(8), 847–851. 24766579 10.1080/17461391.2014.911354

[eph70243-bib-0012] Cowley, E. S. , Olenick, A. A. , McNulty, K. L. , & Ross, E. Z. (2021). “Invisible sportswomen”: The sex data gap in sport and exercise science research. Women in Sport and Physical Activity Journal, 29(2), 146–151.

[eph70243-bib-0013] de Aquino Lemos, V. , Antunes, H. K. M. , dos Santos, R. V. T. , Lira, F. S. , Tufik, S. , & de Mello, M. T. (2012). High altitude exposure impairs sleep patterns, mood, and cognitive functions. Psychophysiology, 49(9), 1298–1306. 22803634 10.1111/j.1469-8986.2012.01411.x

[eph70243-bib-0014] Elliott‐Sale, K. J. , Minahan, C. L. , de Jonge, X. A. J. , Ackerman, K. E. , Sipilä, S. , Constantini, N. W. , Lebrun, C. M. , & Hackney, A. C. (2021). Methodological considerations for studies in sport and exercise science with women as participants: A working guide for standards of practice for research on women. Sports Medicine, 51(5), 843–861. 33725341 10.1007/s40279-021-01435-8 PMC8053180

[eph70243-bib-0015] Erez, D. , Lieberman, H. R. , Baum, I. , Ketko, I. , & Moran, D. S. (2024). Ad libitum caffeine consumption, cognitive performance, and sleep in special forces soldiers during a 96‐h combat exercise. Frontiers in Neuroscience, 18, 1419181. 38975243 10.3389/fnins.2024.1419181 PMC11224469

[eph70243-bib-0016] Giles, G. E. , Mahoney, C. R. , Caruso, C. , Bukhari, A. S. , Smith, T. J. , Pasiakos, S. M. , McClung, J. P. , & Lieberman, H. R. (2019). Two days of calorie deprivation impairs high level cognitive processes, mood, and self‐reported exertion during aerobic exercise: A randomized double‐blind, placebo‐controlled study. Brain and Cognition, 132, 33–40. 30831453 10.1016/j.bandc.2019.02.003

[eph70243-bib-0017] Golja, P. , Kacin, A. , Tipton, M. , Eiken, O. , & Mekjavic, I. (2004). Hypoxia increases the cutaneous threshold for the sensation of cold. European Journal of Applied Physiology, 92(1‐2), 62–68. 14991327 10.1007/s00421-004-1058-9

[eph70243-bib-0018] Hamarsland, H. , Paulsen, G. , Solberg, P. A. , Slaathaug, O. G. , & Raastad, T. (2018). Depressed physical performance outlasts hormonal disturbances after military training. Medicine & Science in Sports & Exercise, 50(10), 2076–2084. 29927875 10.1249/MSS.0000000000001681

[eph70243-bib-0019] Hinde, K. , Low, C. , Lloyd, R. , & Cooke, C. (2018). Interaction Between Ambient Temperature, Hypoxia, and Load Carriage on Respiratory Muscle Fatigue. Aerospace Medicine and Human Performance, 89(11), 952–960. 30352647 10.3357/AMHP.5108.2018

[eph70243-bib-0020] Hinde, K. L. , O'Leary, T. J. , Greeves, J. P. , & Wardle, S. L. (2021). Measuring protein turnover in the field: Implications for military research. Advances in Nutrition, 12(3), 887–896. 33079983 10.1093/advances/nmaa123 PMC8166569

[eph70243-bib-0021] Jafarian, S. , Gorouhi, F. , Taghva, A. , & Lotfi, J. (2008). High‐altitude sleep disturbance: Results of the Groningen Sleep Quality Questionnaire survey. Sleep Medicine, 9(4), 446–449. 17869574 10.1016/j.sleep.2007.06.017

[eph70243-bib-0022] Jung, M. , Zou, L. , Yu, J. J. , Ryu, S. , Kong, Z. , Yang, L. , Kang, M. , Lin, J. , Li, H. , & Smith, L. (2020). Does exercise have a protective effect on cognitive function under hypoxia? A systematic review with meta‐analysis. Journal of Sport and Health Science, 9(6), 562–577. 32325144 10.1016/j.jshs.2020.04.004 PMC7749263

[eph70243-bib-0023] Keramidas, M. E. , Gadefors, M. , Nilsson, L.‐O. , & Eiken, O. (2018). Physiological and psychological determinants of whole‐body endurance exercise following short‐term sustained operations with partial sleep deprivation. European Journal of Applied Physiology, 118(7), 1373–1384. 29687266 10.1007/s00421-018-3869-0 PMC6028900

[eph70243-bib-0024] Killgore, W. D. (2010). Effects of sleep deprivation on cognition. Progress in Brain Research, 185, 105–129. 21075236 10.1016/B978-0-444-53702-7.00007-5

[eph70243-bib-0025] Kolstoe, S. E. , Affleck, P. , Cons, J. , & Davis, M. (2023). Call of duty: The ethical imperative to increase the participation of women in UK military research. BMJ Military Health, 169(1), 102. 35772793 10.1136/military-2022-002168

[eph70243-bib-0026] Lieberman, H. R. , Farina, E. K. , Caldwell, J. , Williams, K. W. , Thompson, L. A. , Niro, P. J. , Grohmann, K. A. , & McClung, J. P. (2016). Cognitive function, stress hormones, heart rate and nutritional status during simulated captivity in military survival training. Physiology & Behavior, 165, 86–97. 27374427 10.1016/j.physbeh.2016.06.037

[eph70243-bib-0027] Lieberman, H. R. , Niro, P. , Tharion, W. J. , Nindl, B. C. , Castellani, J. W. , & Montain, S. J. (2006). Cognition during sustained operations: Comparison of a laboratory simulation to field studies. Aviation, Space, and Environmental Medicine, 77(9), 929–935. 16964742

[eph70243-bib-0028] Lim, J. , & Dinges, D. F. (2010). A meta‐analysis of the impact of short‐term sleep deprivation on cognitive variables. Psychological Bulletin, 136(3), 375–389. 20438143 10.1037/a0018883 PMC3290659

[eph70243-bib-0029] Lloyd, A. , & Havenith, G. (2016). Interactions in human performance: An individual and combined stressors approach. Temperature, 3(4), 514–517. 10.1080/23328940.2016.1189991 PMC5198808 28090553

[eph70243-bib-0030] Lloyd, A. , Hodder, S. , & Havenith, G. (2015). The interactive effect of cooling and hypoxia on forearm fatigue development. European Journal of Applied Physiology, 115(9), 2007–2018. 25963379 10.1007/s00421-015-3181-1

[eph70243-bib-0031] Lynch, A. H. , Norchi, C. H. , & Li, X. (2022). The interaction of ice and law in Arctic marine accessibility. Proceedings of the National Academy of Sciences, 119(26), e2202720119. 10.1073/pnas.2202720119 PMC9245659 35727968

[eph70243-bib-0032] Margolis, L. M. , Murphy, N. E. , Martini, S. , Gundersen, Y. , Castellani, J. W. , Karl, J. P. , Carrigan, C. T. , Teien, H. K. , Madslien, E. H. , Montain, S. J. , & Pasiakos, S. M. (2016). Effects of supplemental energy on protein balance during 4‐d arctic military training. Medicine and Science in Sports and Exercise, 48(8), 1604–1612. 27054679 10.1249/MSS.0000000000000944

[eph70243-bib-0033] Margolis, L. M. , Murphy, N. E. , Martini, S. , Spitz, M. G. , Thrane, I. , McGraw, S. M. , Blatny, J. M. , Castellani, J. W. , Rood, J. C. , Young, A. J. , & Montain, S. J. (2014). Effects of winter military training on energy balance, whole‐body protein balance, muscle damage, soreness, and physical performance. Applied Physiology, Nutrition, and Metabolism, 39(12), 1395–1401. 10.1139/apnm-2014-0212 25386980

[eph70243-bib-0034] Marrao, C. , Tikuisis, P. , Keefe, A. A. , Gil, V. , & Giesbrecht, G. G. (2005). Physical and cognitive performance during long‐term cold weather operations. Aviation, Space, and Environmental Medicine, 76(8), 744–752. 16110690

[eph70243-bib-0035] McMorris, T. , Hale, B. J. , Barwood, M. , Costello, J. , & Corbett, J. (2017). Effect of acute hypoxia on cognition: A systematic review and meta‐regression analysis. Neuroscience & Biobehavioral Reviews, 74(part A), 225–232. 28111267 10.1016/j.neubiorev.2017.01.019

[eph70243-bib-0036] Mesas, A. E. , de Arenas‐Arroyo, S. N. , Martinez‐Vizcaino, V. , Garrido‐Miguel, M. , Fernández‐Rodríguez, R. , Bizzozero‐Peroni, B. , & Torres‐Costoso, A. I. (2023). Is daytime napping an effective strategy to improve sport‐related cognitive and physical performance and reduce fatigue? A systematic review and meta‐analysis of randomised controlled trials. British Journal of Sports Medicine, 57(7), 417–426. 36690376 10.1136/bjsports-2022-106355

[eph70243-bib-0037] Moore, D. C. , Notley, S. R. , Aisbett, B. , & Main, L. (2025). The cumulative effects of consecutive days of prolonged, physical work or activity on heat strain and physical performance: A systematic review. Applied Physiology, Nutrition, and Metabolism, 50, 1–14. 10.1139/apnm-2024-0391 39889274

[eph70243-bib-0038] Nindl, B. C. , Leone, C. D. , Tharion, W. J. , Johnson, R. F. , Castellani, J. W. , Patton, J. F. , & Montain, S. J. (2002). Physical performance responses during 72 h of military operational stress. Medicine and Science in Sports and Exercise, 34(11), 1814–1822. 12439088 10.1097/00005768-200211000-00019

[eph70243-bib-0039] Nindl, B. C. , Rarick, K. R. , Castellani, J. W. , Tuckow, A. P. , Patton, J. F. , Young, A. J. , & Montain, S. J. (2006). Altered secretion of growth hormone and luteinizing hormone after 84 h of sustained physical exertion superimposed on caloric and sleep restriction. Journal of Applied Physiology, 100(1), 120–128. 16141374 10.1152/japplphysiol.01415.2004

[eph70243-bib-0040] O'Leary, T. J. , Young, C. , Wardle, S. , & Greeves, J. (2023). Gender data gap in military research: A review of the participation of men and women in military musculoskeletal injury studies. British Medical Journal Military Health, 169(1), 84–88. 35042757 10.1136/bmjmilitary-2021-002015

[eph70243-bib-0041] Opstad, P. K. , Ekanger, R. , Raabe, N. , & Nummestad, M. (1978). Performance, mood, and clinical symptoms in men exposed to prolonged, severe physical work and sleep deprivation. Aviation, Space, and Environmental Medicine, 49(9), :1065–1073. 697668

[eph70243-bib-0042] Ritland, B. M. , Simonelli, G. , Gentili, R. J. , Smith, J. C. , He, X. , Mantua, J. , Balkin, T. J. , & Hatfield, B. D. (2019). Effects of sleep extension on cognitive/motor performance and motivation in military tactical athletes. Sleep Medicine, 58, 48–55. 31096123 10.1016/j.sleep.2019.03.013

[eph70243-bib-0043] Rognum, T. O. , Vartdal, f. , Rodahl, k. , Opstad, p. K. , Knudsen‐baas, O. , Kindt, E. , & Withey, W. R. (1986). Physical and mental performance of soldiers on high‐and low‐energy diets during prolonged heavy exercise combined with sleep deprivation. Ergonomics, 29(7), 859–867. 3743541 10.1080/00140138608967198

[eph70243-bib-0044] Salmon, O. F. , Segovia, M. D. , Ugale, C. B. , & Smith, C. M. (2023). The impact of cold, hypoxia, and physical exertion on pistol accuracy and tactical performance. Journal of Thermal Biology, 117, 103676. 37738801 10.1016/j.jtherbio.2023.103676

[eph70243-bib-0045] Smith, B. P. , Browne, M. , Armstrong, T. A. , & Ferguson, S. A. (2016). The accuracy of subjective measures for assessing fatigue related decrements in multi‐stressor environments. Safety Science, 86, 238–244.

[eph70243-bib-0046] Ståhle, L. , Ståhle, E. L. , Granström, E. , Isaksson, S. , Annas, P. , & Sepp, H. (2011). Effects of sleep or food deprivation during civilian survival training on cognition, blood glucose and 3‐OH‐butyrate. Wilderness & Environmental Medicine, 22(3), 202–210. 21962046 10.1016/j.wem.2011.02.018

[eph70243-bib-0047] Stott, P. (2016). How climate change affects extreme weather events. Science, 352(6293), 1517–1518. 27339968 10.1126/science.aaf7271

[eph70243-bib-0048] Tipton, M. (2012). A case for combined environmental stressor studies. Extreme Physiology & Medicine, 1(1), 7. 23849435 10.1186/2046-7648-1-7 PMC3710159

[eph70243-bib-0049] Williams, T. B. , Badariotti, J. I. , Corbett, J. , Miller‐Dicks, M. , Neupert, E. , McMorris, T. , Ando, S. , Parker, M. O. , Thelwell, R. C. , & Causer, A. J. (2024). The effects of sleep deprivation, acute hypoxia, and exercise on cognitive performance: A multi‐experiment combined stressors study. Physiology & Behavior, 274, 114409. 37977251 10.1016/j.physbeh.2023.114409

